# Using mHealth to Improve Communication in Adult Day Services Around the Needs of People With Dementia: Mixed Methods Assessment of Acceptability and Feasibility

**DOI:** 10.2196/49492

**Published:** 2024-03-01

**Authors:** Amy Zheng, Marissa Bergh, Komal Patel Murali, Tina Sadarangani

**Affiliations:** 1 New York University Rory Meyers College of Nursing New York, NY United States; 2 Hartford Institute for Geriatric Nursing New York University Rory Meyers College of Nursing New York, NY United States; 3 New York University Grossman School of Medicine New York, NY United States

**Keywords:** adult day services, primary health care, health communication, dementia, mobile health, mHealth, community-based, health care, older adults, older adult, chronic condition, health information, feasibility, acceptability, CareMOBI, mixed methods design, caregivers, caregiver, care workers, nurses, social workers

## Abstract

**Background:**

Adult day services (ADS) provide community-based health care for older adults with complex chronic conditions but rely on outdated methods for communicating users’ health information with providers. CareMOBI, a novel mobile health (mHealth) app, was developed to address the need for a technological platform to improve bidirectional information exchange and communication between the ADS setting and providers.

**Objective:**

This study aims to examine the feasibility and acceptability of CareMOBI in the ADS setting.

**Methods:**

A concurrent-triangulation mixed methods design was used, and participants were client-facing ADS staff members, including direct care workers (paid caregivers), nurses, and social workers. Interviews were conducted to describe barriers and facilitators to the adoption of the CareMOBI app. The acceptability of the app was measured using an adapted version of the Technology Acceptance Model questionnaire. Data were integrated into 4 themes as anchors of an informational matrix: ease of use, clinical value, fit within workflow, and likelihood of adoption.

**Results:**

A mix of ADS staff (N=22) participated in the study. Participants reported high levels of acceptability across the 4 domains. Qualitative findings corroborated the questionnaire results; participants viewed the app as useful and were likely to implement CareMOBI in their practice. However, participants expressed a need for proper training and technical support throughout the implementation process.

**Conclusions:**

The CareMOBI app has the potential to improve care management in the ADS setting by promoting effective communication through an easy-to-use and portable method. While the integration of CareMOBI is acceptable and feasible, developing role-specific training modules and technical assistance programs is imperative for successful implementation within the ADS setting.

## Introduction

Adult day services (ADS), commonly referred to as adult day care, is a vital but overlooked source of health and social care for the burgeoning population of older adults with complex chronic conditions—particularly those with Alzheimer disease and related dementia (ADRD) [1]. ADS sites are nonresidential, congregate, and community-based facilities that offer interdisciplinary services, including opportunities for socialization, nursing care, and nutritious meals for chronically ill or functionally impaired adults. Each day in the United States, 251,100 adults with complex health and social needs receive care in the ADS setting: 65% have some combination of ADRD, diabetes, depression, heart disease, or other chronic conditions; 72% live below federal poverty lines; and 55% are from a racial or ethnic minoritized group [2]. Clients attend 2 to 5 days per week and may receive assistance with personal care, physical therapy, vital sign monitoring, and medication administration, while also participating in organized group activities [3]. ADS staff are skilled at using their in-depth, serial observations of clients to identify warning signs of acute illness and promote early clinical intervention, which are especially important in persons with ADRD, who may not be able to identify or communicate changes in their health status.

However, ADS sites face numerous barriers in communicating concerning changes in health status to primary care providers [4]. Ineffective communication in health care has been associated with costs exceeding US $10 billion, in addition to adverse outcomes and increased mortality [5,6]. We previously found that communication between free-standing community-based ADS centers is hampered by reliance on antiquated methods of information exchange that challenge information sharing and care coordination [7]. When ADS clients experience acute changes in health status or behavior, information is typically reported to primary care providers through fax or voicemail messages, which often results in nonresponse, delayed diagnosis, referral or treatment, and inadequate follow-up. Primary care providers and ADS staff have agreed that communication between them is infrequent, delayed, incomplete, and unreliable. Primary care providers prefer to communicate using direct messaging systems within their electronic health record; however, 92% of ADS sites in the United States lack the resources for interoperable electronic health record systems that enable e-communication [8]. The lack of resources shifts the burden of communication between ADS sites and primary care to the family caregiver (herein referred to as caregivers) who must deliver the information from the ADS site to the primary care provider at the point of service. Although engaged caregivers are often willing to track medical information and coordinate care, they may lack the necessary time, resources, and health education, resulting in medical errors or delays in care.

**Figure 1:**
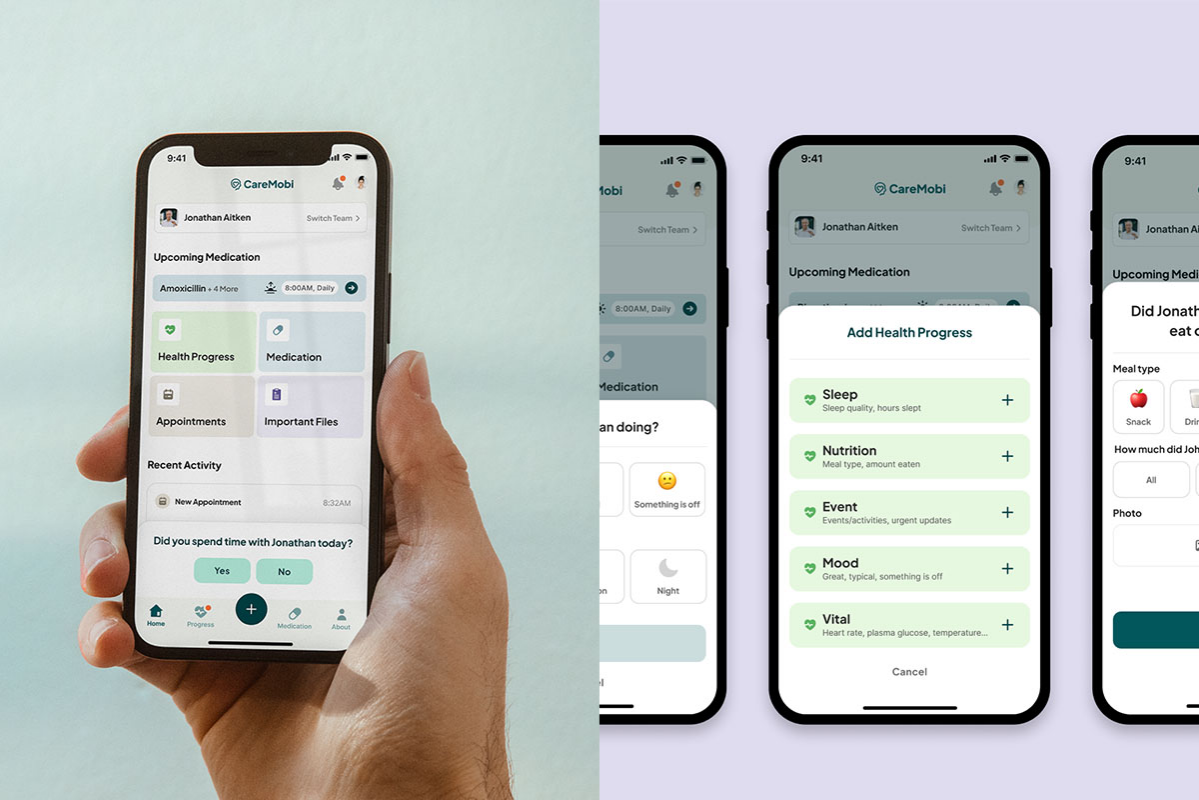
The home page of the CareMOBI mobile app and select features.

We developed CareMOBI (mobile health for organizations to bolster interconnectedness), a mobile health (mHealth) app prototype, to address the consistent need for improved care coordination and communication among care team members supporting care in home and community settings (Figure 1). CareMOBI acts as a centralized hub for families to track and share information about their loved one’s day-to-day health with other care team members (ie, ADS staff, home health aides, and family members). This enables multiple caregivers to support an individual across home and community-based settings. Within CareMOBI, individuals are first invited to a person’s care team. Each member can then record information and share updates about a person’s health, including how they ate, slept, or felt on a given day. They can track vital signs, keep up-to-date medication lists, and track medication administration. They can also track appointments and make notes of observations or questions to ask providers. Most importantly, care team members can report and be alerted when an individual is exhibiting concerning symptoms or experiencing an emergency. Information entered with the app can be summarized and shared via a PDF file to support shared clinical decision-making at appointments or appended to a chart. CareMOBI is a low-cost, portable means of exchanging information between ADS sites, caregivers, and health care providers. It also provides a centralized platform that allows ADS staff, caregivers, and care providers to provide updates and track the health progress of a person who cannot do so independently, as well as share any urgent concerns and observations, such as new or worsening confusion, behavioral changes, or abnormal vital signs. These features are designed to support critical early identification of clinical issues with the goal of reducing costly, traumatic, and avoidable emergency department care or hospitalizations, as well as overall care management for people with complex care needs. The purpose of this mixed methods study was to examine the feasibility and acceptability of CareMOBI in an ADS setting through surveys and interviews with ADS staff.

## Methods

We used a mixed methods concurrent triangulation design to (1) assess acceptability and feasibility of the CareMOBI prototype among adult day center staff and (2) identify factors contributing to eventual likelihood of adoption or nonadoption.

### Setting and Sample

Participants were eligible if (1) they were paid employees of a participating adult day center and (2) they had a client-facing role that involved daily interaction with persons living with dementia, as these are the target end users of the app in the ADS setting. Examples of client-facing staff are registered nurses, social workers, and program assistants. Individuals were excluded if they had worked in their current position for less than 6 months. Purposive sampling was used to recruit a diverse multistakeholder sample that represented the range of professionals in adult day centers (eg, registered nurses, social workers, and program directors).

### Ethical Considerations

Eligible staff members were identified with the help of administrators at participating adult day centers. A research assistant contacted them by email or phone according to their preference, described the study, confirmed participants’ eligibility, and subsequently obtained informed consent. In total, 22 staff members from adult day centers in 3 states (New York, California, and Georgia) enrolled. All enrollees received a US $50 gift card for their participation. The New York University committee on activities involving human subjects provided Institutional Review Board approval for this study (IRB-FY2020-4615).

### Procedures

Data collection consisted of one-to-one semistructured interviews and the completion of the Technology Acceptance Model questionnaire, adapted for health care settings [9]. The Technology Acceptance Model was developed 4 decades ago to explore factors that shape workers’ intention to use emerging technology. Rooted in the theory of reasoned action, it explores the beliefs and norms that influence attitudes and expectations that increase the desire to carry out a behavior. Thus, it is a logical and established framework to explore factors determining the likelihood of adoption of CareMOBI in ADS. The Technology Acceptance Model questionnaire provided insight into the acceptability of the app, while subsequent qualitative interviews provided additional insight into concerns around the feasibility of embedding CareMOBI within ADS.

Approximately 1 week prior to the interview, participants received a confirmation email that contained a link to an interactive prototype of CareMOBI. The prototype could be accessed from a smartphone, tablet, or computer. Participants were instructed to watch a 2-minute informational video about the app, also linked in the email, and were then asked to spend approximately 10 minutes navigating through the interactive prototype to complete several relevant tasks, including logging in, adding a new medication, recording day-to-day activities of a typical person living with dementia at their adult day center, as well as any health-related progress notes. They were also asked to use filters within CareMOBI to locate information about the person living with dementia in whom they were interested.

### Qualitative Data Collection and Analytic Procedures

Web-based interviews were scheduled based on participants’ availability and conducted via a secure web-based platform. UX (user experience) and UI (user interface) design professionals, who had extensive knowledge of user-testing, provided input to develop a semistructured interview guide at the product development firm that built the CareMOBI prototype.

Participant interviews lasted 30 minutes on average. Interviews were conducted by either the principal investigator (TS) or a trained research assistant. Both individuals have extensive experience with qualitative interviewing. Open-ended questions allowed participants to elaborate on their reaction to the CareMOBI app and allowed the researchers to elicit information on different factors influencing their perceptions of the app, aspects related to its usability, and potential feasibility issues, including workflow integrations. Sample questions included:

1. What is your overall impression of the app?
2. In what ways do you think this app could help address some of the challenges you face in organizing and communicating information around the needs of person living with dementia?
3. Are there other challenges in caring for person living with dementia that this app doesn’t address? What might those be?
4. What features in the app were most confusing for you to understand/find/utilize?

All interviews were recorded, professionally transcribed, and reviewed for accuracy. Field notes by the interviewer supplemented the tape-recorded interviews. A detailed audit trail documented the rationale for any methodological changes during the interview or analysis (ie, when unique follow-up questions were posed).

Qualitative data were analyzed using a content analysis approach. A preliminary codebook was developed a priori based on the interview guide as a coding scheme for all transcripts. The research team discussed any text that could not be categorized within the codebook to determine if a new category or code needed to be defined or aligned with an existing category or code. The codebook was continuously updated to reflect the iterative process. Initially, 2 coders coded independently in Dedoose, a web-based platform for qualitative and mixed method coding, and met weekly to review coding and resolve any disagreements. To ensure the reliability and consistency of coding, a third independent coder analyzed a subset of 20% (n=5) of the transcripts. The principal investigator addressed any unresolved disagreements, as well as potential new categories or codes, in team meetings. Codes were summarized within cases and then compared across cases to identify emerging themes. Saturation occurred when no new themes emerged. The research team members regularly debriefed to discuss and validate the results of the analysis. Quotations were provided with participant’s initials to maintain anonymity.

### Quantitative Data Collection and Analytic Procedures

Upon finishing the interview, participants completed a web-based questionnaire. In addition to providing basic demographic information, participants completed an adapted version of the Technology Acceptance Model questionnaire, which was previously validated for use in health care settings [9]. Responses to the 33 survey items enabled further examination of factors that could influence the eventual adoption of CareMOBI, and the anonymous nature reduced the potential for social desirability bias. Respondents rated each item on a 7-point Likert scale ranging from “strongly disagree” to “strongly agree.” Each domain-specific question was averaged to determine scores. Higher scores corresponded to higher perceived acceptability.

Descriptive statistics were used to characterize the sample, with measures of central tendency and spread for continuous measures and frequencies and percentages for dichotomous or categorical variables. Calculations for quantitative statistics were done using Qualtrics software (version May 2023; Qualtrics LLC).

### Integration of Qualitative and Quantitative Data

Qualitative and quantitative data were integrated in the third and final phase of analysis. We sought to align with the Technology Acceptance Model: perceived ease of use, perceived value in clinical care, fit within existing workflows, and end users’ overall likelihood of adoption. Using the 4 themes as anchors, we developed an informational matrix in which qualitative data were embedded and compared with quantitative data. Using triangulation methods, we sought to understand the overall likelihood of adoption of the app by end users (quantitatively) and factors underpinning this across cases within each stakeholder group (qualitatively).

## Results

### Overview

The primary goals of this study were to (1) assess the acceptability of the CareMOBI prototype among adult day center staff and (2) identify factors contributing to the eventual likelihood of adoption or nonadoption. We evaluated the feasibility and acceptability of CareMOBI in an ADS setting quantitatively and qualitatively according to 4 themes: perceived ease of use, perceived value in clinical practice, how the mHealth app fits within existing workflows, and likelihood of adoption.

### Study Sample

The total sample (N=22) of ADS staff members was majority non-Hispanic (20/22, 91%) and White (16/22, 73%). Most participants (19/22, 86%) were aged between 40 and 69 years. All respondents (100%) identified as women. Among those who responded when asked about their role, the majority (10/18, 56%) identified as direct care workers or professional caregivers (aides) in adult day centers (see Table 1).

**Table 1:** Demographic characteristics of adult day center staff.

Participant characteristics		Participants (N=22), n (%)
**Gender**		
	Women	22 (100)
**Race**		
	African American or Black	5 (23)
	White	16 (73)
	Other	1 (5)
**Ethnicity**		
	Hispanic or Latino	2 (9)
	Not Hispanic or Latino	20 (91)
**Age group (years)**		
	≤29	2 (9)
	30-39	0 (0)
	40-49	9 (41)
	50-59	5 (23)
	60-69	5 (23)
	≥70	1 (5)
**Education**		
	High school graduate, or equivalent	1 (5)
	Professional degree	2 (9)
	Some college credit, no degree	4 (18)
	Associate’s degree	4 (18)
	Bachelor’s degree	5 (23)
	Master’s degree	5 (23)
	Doctorate degree	1 (5)
**Role at adult day center**		
	Direct care worker or professional caregiver	10 (44)
	Registered nurse	2 (9)
	Social worker	1 (5)
	Program director	4 (18)
	Activities coordinator	1 (5)
	Preferred not to answer or did not specify	4 (18)

### Perceived Ease of Use

**Figure 2:**
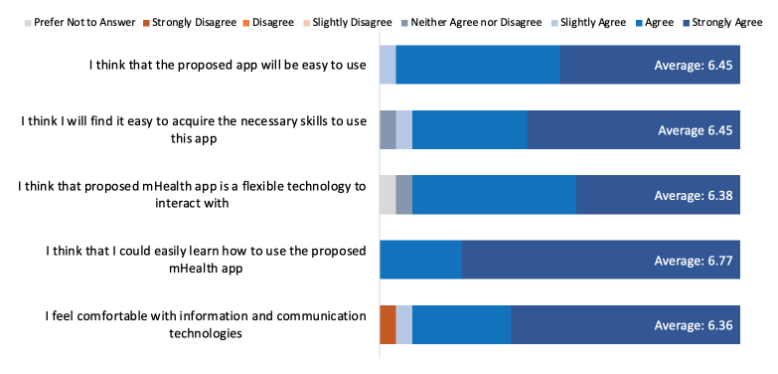
Quantitative survey answer distributions for perceived ease of use. mHealth: mobile health.

Perceived ease of use describes the effort level associated with understanding and using CareMOBI. There were 5 questions within the Technology Acceptance Model questionnaire that assessed the perceived ease of use: the overall ease of use, the technological skill required to use the app, and the user’s comfort level with the app (Figure 2). The mean score for this domain was 6.48, indicating a high ease of use for CareMOBI. The question with the highest mean score was “I think that I could easily learn how to use the proposed mHealth app” (mean of 6.77 or strongly agree). The lowest scoring question was “I feel comfortable with information and communication technologies” (mean of 6.00 or agree).

The qualitative interviews provided insight into what aspects of CareMOBI facilitated the ease of use for the participants (Table 2). Participants reported that the organized layout was simple and easy to navigate. Many expressed appreciation for how they did not have to spend time searching for what they needed, and that all of the features were “at their fingertips” (ADC-LP). However, the qualitative interviews did reveal that while ADS staff found CareMOBI easy to use, they had concerns about family caregivers’ ability to use it. This included challenges with the functionality of some app features and the lack of non-English language options for caregivers and patients with low English proficiency. One respondent stated:

For those who don’t speak English, that’s another [issue]—because participants here, we have different languages spoken here, more than five, six, seven languages. For caregivers who don’t understand or don’t speak English well, that’s challenging as well.

ADC-AL

Furthermore, some participants expressed concern that technology-based solutions may not be appropriate methods of communication for some clients due to low technology proficiency and preferences for traditional methods. While many of the features were easy to use, participants noted a few exceptions that were too complex and potentially confusing for them to navigate, particularly the appointment tracking feature. A staff member noted that while navigating the app was “pretty easy,” it might confuse caregivers who could “get mixed up on appointments, whether it’s outside or they’re coming here” (ADC-NF).

**Table 2:** Qualitative subthemes for perceived ease of use.

Qualitative subthemes	Qualitative feedback
User centered design	“I liked it. I thought it was well organized. I like where everything’s at your fingertips. You can do it on your phone and share it with who you’d like to share it with. I was pretty impressed.” [ADC-LP]
Functionality	“It’s not letting me click on event. I think this was the issue before. I had to go all the way out to—and come back in if I wanted to access something other than—so, I’m stuck on mood right now. I can go to vitals. I can go to all, but I can’t go to event.” [ADC-DT]
Noninclusive Features	“Additional to that, for those who don’t speak English, that’s another—because participant here, we have different languages spoken here, more than five, six, seven languages. For caregiver who don’t understand or don’t speak English well, that’s challenging as well.” [ADC-AL]
Confusing or overly complex features	“...those boxes are right there in the forefront, so it’s pretty easy, but it’s easy to get mixed up on appointments, whether it’s outside or they’re coming here...” [ADC-NF]

### Perceived Value in Clinical Practice

**Figure 3:**
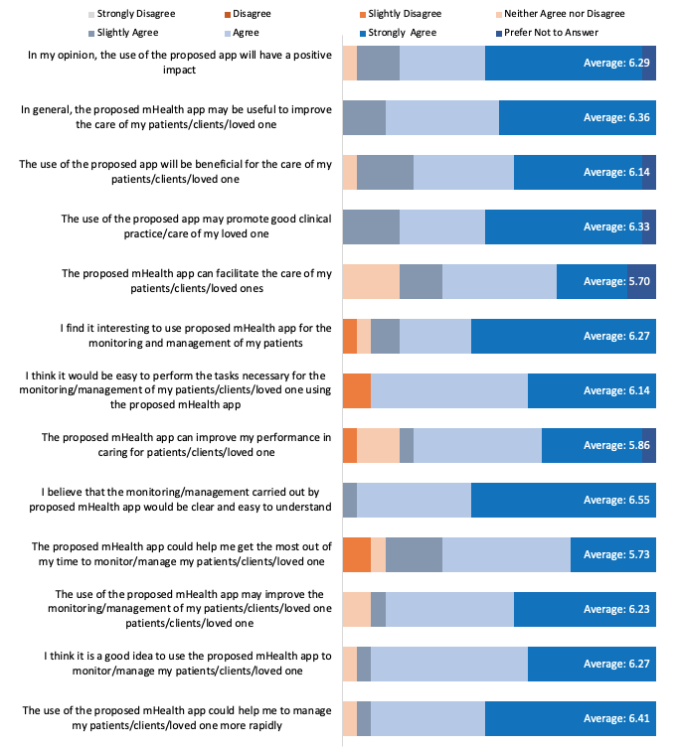
Quantitative survey answer distributions for perceived value in clinical practice. mHealth: mobile health.

Perceived value in clinical practice is the degree to which CareMOBI could improve patient care and management within ADS. Thirteen questions in the survey assessed this domain (Figure 3). The mean score for this domain was 6.18 out of 7, indicating that participants generally rated the CareMOBI app well in terms of potential value to their clinical practice. The item with the highest average score was “The proposed mHealth app can facilitate the care of my patients/clients/loved ones” (mean of 6.55 or strongly agree). The item with the lowest average score was “The proposed mHealth app can facilitate the care of my patients/clients/loved ones” (mean of 5.70 or agree).

Data from the qualitative interviews suggested that staff felt there was immediate value to implementing the app in the day center (Table 3).

I know how it could help me and my family, so it just lights the torch for me the more. This is so huge because the next wave, they’re gonna be—they’re more technology savvy, so they’re gonna be wanting a lot of this information more so, so we have to keep evolving to be able to help make sure that we are communicating right away and training our staff to use it. Both the people were so excited, like, “Oh, my God, it’s so much easier,” and then they get an alert. I’m like, “Yeah, just to get an alert.”

ADC-NF

Several features of the CareMOBI app would improve the staff’s ability to manage care for ADS participants. Specifically, study participants appreciated the alert and notifications feature for caregivers and staff and the built-in features that allow easy “communication on a daily basis” for members of the care team. The CareMOBI app has the capacity to track health progress (ie, vital sign trends, medication management, and appointment reminders) beyond ADS through caregiver and health professional portals. These features were viewed as beneficial to augment the traditional emergency communications and to facilitate chronic disease management within the adult day centers. While most features received positive feedback, 1 staff participant noted that the behavioral assessment tool, which used emoticons (or “smiley faces”) to evaluate daily behavior was oversimplified. Generally, both the qualitative and quantitative data indicate that staff participants perceived the CareMOBI app as a potentially valuable clinical tool to augment their practice due to its ability to facilitate communication, consolidate patient information, and organize care management.

**Table 3:** Qualitative subthemes for perceived value in clinical care.

Subthemes	Qualitative feedback
Patient monitoring and management	“I know how it could help me and my family, so it just lights the torch for me the more. Both the people were so excited, like, ‘Oh, my God, it’s so much easier,’ and then they get an alert. I’m like, ‘Yeah, just to get an alert.’” [ADC-NF]
Real time communication among the care team	“I think that the real-time communication makes it useful. What we’re seeing on a daily basis, if we’re able to communicate on a daily basis as opposed to—right now, we have—we will call a family member if there is an acute episode. If there’s not an acute episode, we’re not gonna just be like, ‘Hey, this is what happened today. This is what we saw. Blah, blah, blah, blah, blah.’” [ADC-DT]
Updates on health progress	“Even though they might not be able to communicate on a consistent basis when I call, tell me what was happenin’ this week or this weekend. I could at least gather that information and, like I said, with the app there, I could say, ‘Per a neighbor who’s slash the caregiver, this is what they observed.’” [ADC-LSM]
Trends	“Then visually having the client on-site, it would be able to give me a better way of communicating, ‘Oh, yeah. This is not just a bump. [Laughter] It’s a big bump from the baseline.’ That’s the whole point, being able to look at the trend.” [ADC-LSM]
Assist caregivers	“We, at adult daycare center, we take care of not only participant, but the caregiver also. When caregiver’s wellbeing are good, our participant are good. They can have quality of life at home instead of ending up in a placement. It’s not their wishes and their goals. That’s very good that in the app.” [ADC-AL]
Schedules and reminders	“I think it’s very good to have [appointments] on the calendar. It’s forgetful for those who have cognitive impairment and caregiver busy with the schedule. When you have those in place, it’s very good for the keep ongoing with the appointment, with the medication, make sure they take medication on time, don’t miss any doses. That help both caregiver and participant to do the daily tasks appropriately.” [ADC-AL]
Patient profile information	“Then they sign up their loved one and their pictures, information about themselves, information about their loved one, the medication they’re on, what they, little bit about themselves, a little bit about their loved one’s background.” [ADC-EK]
Disliked features	“Sometimes behavior issues where someone is having extraordinary anxiety or—then I think that that might be able to be addressed. It would be challenging to properly do that with just the smiley faces, I think. I thought that was very surface-related, very cursory thing. I’m not sure how incredibly helpful that would be unless there’s some depth later somewhere. That would be my only concern about that.” [ADC-HK]
Health progress logs	“I found that it had, the activities part of it and how their morning started or how they slept, and things that I deal with hands on here.” [ADC-LR]
Miscellaneous ways the app enhances care	“That is perfect, because then—in outcomes for us or if we utilize it regularly, it would help us with data collection too.” [ADC-BT]

### Fits Within the Workflow

**Figure 4:**
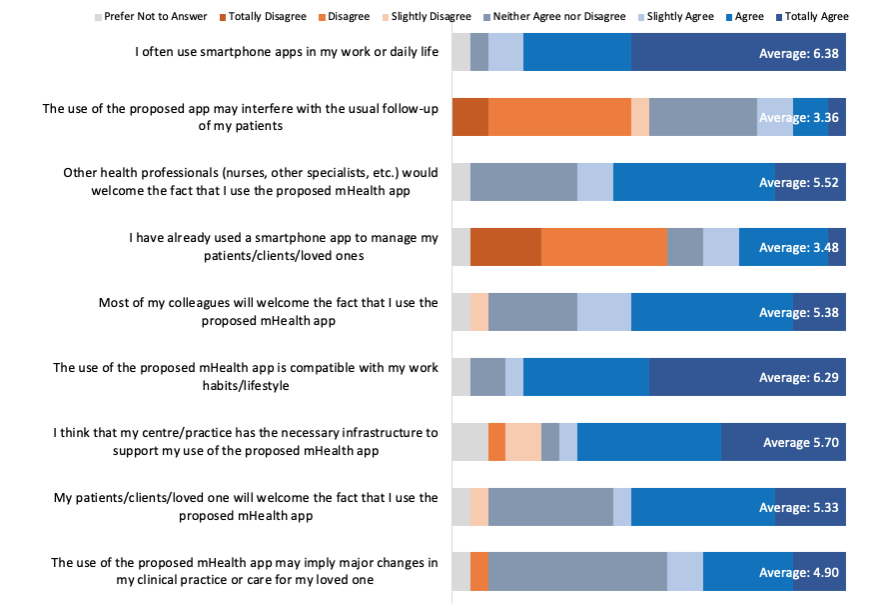
Quantitative survey answer distribution for how CareMOBI fits within the adult day service workflow. mHealth: mobile health.

New technology should be compatible with the existing workflow of staff and patients and cause minimal disruptions to optimize adoption. A total of 9 questions assessed the extent to which the CareMOBI app could integrate into the existing health records and workflow of ADS staff (Figure 4). The mean domain score was 5.14 out of 7, indicating that most participants agreed that the CareMOBI app would fit within the workflow. The item with the highest average score was “I often use smartphone apps in my work or daily life” (mean of 6.38 or strongly agree), and the item with the lowest average score was “The use of the proposed app may interfere with the usual follow up of my patients” (mean of 3.36 or slightly disagree). Due to the wording of this item, a low rating has a positive implication for the compatibility of the CareMOBI app with ADS staff workflow.

However, data from the qualitative interviews highlighted several caveats that could preclude the successful integration of the CareMOBI app into the current staff workflow (Table 4). Staff recommended prioritizing the interoperability of CareMOBI with existing electronic health record systems (eg, TurboTAR) to avoid duplicitous documentation and streamline their workflow. Congruent with the quantitative data, staff participants cited that the lack of existing mHealth apps used in their routine workflows meant that staff and caregivers were not familiar with this interface and could be problematic when initially integrating the app into use. To ameliorate this, the staff participants emphasized the need for sufficient training using videos and other methods for both staff and caregivers, as many described being intimidated by the complexity of emerging technologies. Participants (ADC-JL) also voiced concern that documentation in the CareMOBI app would just be “one more thing” that needs to be done and believed it could be challenging to “actually utilize it” given their other responsibilities. Many participants also imparted that the benefits provided by improved e-communication and information sharing could encourage integration into their workflows. One staff member stated that the app was “great [for] communication with physicians” and another remarked that the ability to be able to take pictures of medication bottles and export patient information “clears up so much confusion” for them. Both the qualitative and quantitative data show relative support for using the app in the workflow but the qualitative interview data reveal several barriers that could inhibit integration.

**Table 4:** Qualitative subthemes for how CareMOBI fits within the adult day service workflow.

Subthemes	Qualitative feedback
Benefits of e-communication	“I actually think it would help a lot, just because it would keep you—it would keep the family more informed, ’cause nowadays everybody uses their cell phones, so it would be right at—right in the palm of their hand. They wouldn’t have to worry about goin’ to their computer. I feel like it would be a great communication with the physicians, letting them know how our members are doing. Also, communication between the staff. Yeah, I thought it was a great tool. I work in another area with home care, and I was thinking how that would work out with that one really well, too. It just kinda would be a great communicator.” [ADC-BW]
Information sharing	“Also, for the medications where they’re seen, that’s huge ’cause they don’t know all the time, the majority of the time. Waiting for a doctor’s office to just snap that picture of the bottle, and the RN gets it right away, it clears up so much confusion.” [ADC-NF]
Suggestions for integrating within existing workflows	“The interoperability, I think that’s the biggie. That we’re entering information one time only so that once you do have your prototype, and it’s down pat, how it maps to the software and, I would say, specifically, TurboTAR ’cause I think it is the most broadly used in adult day healthcare in California, that whatever terminology you’re using on your app maps to sections within TurboTAR so that that’s automatically populating.” [ADC-DT]
Training caregivers to use app	“This is so huge because the next wave, they’re gonna be—they’re more technology savvy, so they’re gonna be wanting a lot of this information more so, so we have to keep evolving to be able to help make sure that we are communicating right away and training our staff to use it.” [ADC-NF]
Feasibility of using app in practice	“I think your issue and hurdle is gonna get people to actually utilize it, especially on the health care side because we already have a thousand things to document and a thousand things to click and a thousand buttons to do. This would just be one more thing. You said something I—you could put an event in like an urgent situation, but would the caregiver still understand that I’m not sitting here with my cell phone watching for something like that to happen so that they’re still responsible for the care of the member. They’re still responsible for calling 911 in any situation like that.” [ADC-JL]

### Likelihood of Adoption

**Figure 5:**
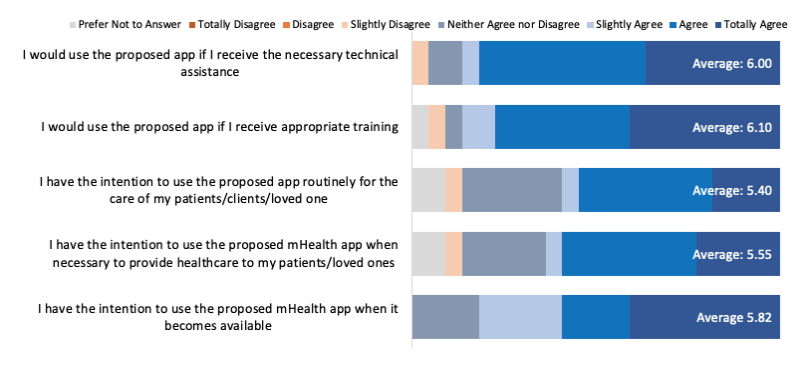
Quantitative survey answer distribution for overall likelihood of adoption of CareMOBI. mHealth: mobile health.

This domain assesses the extent to which staff participants intended to include CareMOBI in their program and use it with clients and caregivers. Five items assessed this domain (Figure 5) and had a mean score of 5.77, a score that suggests most respondents agreed that they intended to use the app after its release. The highest rated item was “I would use the proposed app if I receive appropriate training” (mean of 6.10 or strongly agree) and the lowest rated item was “I have the intention to use the proposed app routinely for the care of my patients/clients/loved one” (mean of 5.40 or slightly agree).

The qualitative data revealed that many staff participants were motivated to adopt the CareMOBI app (Table 5). One participant said, “we were already talking about how we would utilize it in our program,” reflecting the high quantitative score for the likelihood of adoption. The app was designed to reflect the interdisciplinary nature of ADS, and staff participants were appreciative of this aspect. One participant (ADC-LR) commented that they were “very impressed with it and how it touched on all of the different disciplines that we have to do with in an adult day health center, social work, health, activities.” To increase the likelihood of adoption, staff participants also suggested new features of the app to improve care and user experience, including the option to print reports and collect education-level data from patients to help allocate activities.

**Table 5:** Qualitative subthemes for overall likelihood of adoption of CareMOBI.

Subthemes	Qualitative feedback
Overall high likelihood of adoption	“Yep, yep. Very much. I definitely see for those that will—...and I...my co-director, so we were already talking about how we would utilize it in our program. By creating direction and maybe even assistance with family members and setting it up.” [ADC-BT]
Suggested additional features	“I think also, education level. That would help because we do a lot of brain work. If we need to know their education level as to which kind of work to give them. I think education level would be a good question too.” [ADC-EK]
General impression of the app	“I was very impressed with it and how it touched on all of the different disciplines that we have to do with in an adult day health center, social work, health, activities. Yeah, I touched on everything, so I was really impressed with that.” [ADC-LR]

## Discussion

### Principal Findings

The results of this study show that the CareMOBI app is highly acceptable to ADS staff, but there were questions about the feasibility of implementation. The high scores in all 4 domains of the survey—perceived ease of use, perceived value in clinical care, fit within existing workflows, and end users’ overall likelihood of adoption—demonstrate that ADS staff members generally considered that the app was easy to use; added clinical value; fit into their workflow; and that if given the option, would likely adapt the app into their system.

This high receptivity and likelihood of adoption may be attributable to the participatory design process, which engaged staff, caregivers, and providers to identify barriers to and facilitators of effective communication within the ADS setting. Prior to developing the app, members of the study team conducted qualitative interviews nationwide to identify facilitators of effective communication between ADS sites and primary care providers. Results indicated that ADS staff wanted to exchange information bidirectionally with other care team members. They sought technology that would reduce their reliance on fax and voicemail while enabling them to share serial observations of a person living with dementia’s day-to-day health with all members of the care team (eg, ADS staff, health care providers, and caregivers) [7].

In this study, qualitative interviews largely qualified the high scores from survey data; however, interviewees expressed practical concerns about the app’s feasibility, particularly about its fit within the existing workflow of ADS. The feasibility of CareMOBI was threatened by the added workload and documentation burden it has the potential to create, especially for overburdened providers who “already have a thousand things to document and a thousand things to click and a thousand buttons to do.” This concern is not limited to the ADS setting; other research has shown that increased documentation burden in hospitals is a contributing factor to clinician burnout [10,11]. Notably, cost was not mentioned by any of the participants as a concern or barrier to adoption. This is likely because, with low-cost subscriptions (<5 US $/month) and little to no investment overhead, mHealth apps are reputed as a cost-effective solution to meet the needs of complex patients.

The potential workflow disruption and incompatibility with existing technology infrastructures have been cited as one of the major barriers to successful eHealth implementation in a systematic review, and our study was no exception [9,12]. As another participant pointed out, “The interoperability, I think that’s the biggie. That we’re entering information one time only.” This could be mitigated by making CareMOBI interoperable with ADS billing software which is what most sites rely on for day-to-day practice management. In a future study, a back-end dashboard for CareMOBI will be designed to integrate with existing ADS practice management systems, like TurboTAR. This will avoid what 1 participant referred to as “double documentation.”

Another key priority for future development will be the translation of CareMOBI into different languages. ADS is the most diverse subset of long-term care, with nearly 60% of participants identifying as a racial or ethnic minoritized individual [13]. Studies show that ADS can deliver relevant, person-centered care to diverse populations [3]. Adaptations of CareMOBI into other languages will be necessary to improve adoption and inclusivity and ensure the technology reflects the population it is designed to serve.

Successful adoption of new technology in health care settings requires buy-in from staff. It is, therefore, imperative that technology adds clinical value and does not hinder efficiency. While barriers and facilitators of app use in certain health care settings have been well documented, our study is the first to explore the unique perspectives of staff in ADS settings. Resourcing this setting with low-cost mobile technology is especially innovative because ADS sites provide essential care to persons living with dementia but are often resource-constrained and rarely embedded within health care systems.

CareMOBI was developed as a reaction to the overwhelming sentiment that methods of communication in ADS need to be modernized and streamlined to provide optimal care for their clients. Overall, our results from prototype testing show a high level of acceptability among ADS staff with a need for greater attention to feasibility. Recognized by many of the study’s participants, CareMOBI’s biggest strength is its ability to bring all the members of the care team—ADS staff, caregivers, patients, and health care providers—to the same digital table, managing the complex care of older adults as a true team. Previous research by the Agency for Health Care Research and Quality found that care models emphasizing collaborative care were the only effective, evidence-based care models for persons with dementia [14]. By emphasizing effective communication, CareMOBI, with ongoing refinement and enhancement, has significant potential to help streamline and improve care for participants with dementia and other chronic conditions.

### Limitations

While this study is the first to our knowledge to document the perceived feasibility and acceptability of a mHealth app in the ADS setting, these results should be interpreted with some limitations. The participants of this study were only furnished with a prototype of the CareMOBI app with a limited set of capabilities. At times, this elicited frustration from the participants due to certain functions not working correctly. Conversely, testing with only a prototype precludes assessment of real-world functionality, particularly in the assessment of integration within the workflow. This study consisted of a relatively small, sample of exclusively women participants representing 3 states. Regulations and documentation requirements for ADS and other community-based services differ greatly between states, and preferences around the design and use of technology can be influenced by gender [15]. These findings, therefore, may therefore not be generalizable. A larger, more representative study sample would have potentially offered more robust results. However, these findings—the emphasis on minimizing disruptions to workflow, training, and institutional support—are consistent with other previous implementation studies in a broad array of health care settings [9,12,14].

### Future Directions

The feedback gleaned in this study will guide future improvements to the CareMOBI app and on-site implementation of the app. Prior to implementation, the CareMOBI team will aim to develop interactive training modules personalized for each type of end user (ie, a caregiver-specific training module vs an ADS staff or nurse training module). Small-scale implementation studies will help develop a robust framework for successful CareMOBI adoption in ADS settings. Further technological development will be pursued to integrate CareMOBI’s interface with other prominent technologies, like TurboTAR, that are already required in ADS settings.

### Conclusions

This mixed methods study assessed the feasibility and acceptability of ADS staff toward adopting the CareMOBI app in ADS settings. Overall, staff participants were motivated to integrate CareMOBI into their clinical settings because of its potential to ameliorate long-standing communication challenges between ADS staff and other key members of the care team. However, the qualitative interviews highlighted many important potential barriers to successful implementation that researchers will have to consider as they move forward to the next phase of CareMOBI’s development. The CareMOBI team will continue to leverage this important feedback to develop an app that is optimized to fit the needs of all end users and help bring ADS communication into the 21st Century.

## Abbreviations

ADRD: Alzheimer disease and related dementia
ADS: adult day services
mHealth: mobile health
UI: user interface
UX: user experience

## References

[1] Oliver RE, Foster M (2013). Adult day care: an important long-term care alternative and potential cost saver. Mo Med.

[2] Lendon JP, Singh P (2021). Adult day services center participant characteristics: United States, 2018. NCHS Data Brief.

[3] Sadarangani TR, Murali KP (2018). Service use, participation, experiences, and outcomes among older adult immigrants in American adult day service centers: an integrative review of the literature. Res Gerontol Nurs.

[4] Ruggiano N, Brown EL, Fortuna KL (2018). Adult day service providers: untapped potential for care coordination. Nurs Health Sci Res J.

[5] Agarwal R, Sands DZ, Schneider JD (2010). Quantifying the economic impact of communication inefficiencies in U.S. hospitals. J Healthc Manag.

[6] Shrank WH, Rogstad TL, Parekh N (2019). Waste in the US health care system: estimated costs and potential for savings. JAMA.

[7] Zhong J, Boafo J, Brody AA, Wu B, Sadarangani AT (2022). A qualitative analysis of communication workflows between adult day service centers and primary care providers. J Am Med Inform Assoc.

[8] Tables on use of electronic health records and health information exchange among adult day services centers and residential care communities from the 2016 National Study of Long-Term Care Providers. Centers for Disease Control and Prevention.

[9] Gagnon MP, Orruño E, Asua J, Abdeljelil AB, Emparanza J (2012). Using a modified technology acceptance model to evaluate healthcare professionals' adoption of a new telemonitoring system. Telemed J E Health.

[10] Gesner E, Dykes PC, Zhang L, Gazarian P (2022). Documentation burden in nursing and its role in clinician burnout syndrome. Appl Clin Inform.

[11] Gesner E, Gazarian P, Dykes P (2019). The burden and burnout in documenting patient care: an integrative literature review. Stud Health Technol Inform.

[12] Police RL, Foster T, Wong KS (2010). Adoption and use of health information technology in physician practice organisations: systematic review. Inform Prim Care.

[13] Sengupta M, Lendon JP, Caffrey C, Melekin A, Singh P (2022). Post-acute and long-term care providers and services users in the United States, 2017-2018. Vital Health Stat 3.

[14] Ross J, Stevenson F, Lau R, Murray E (2016). Factors that influence the implementation of e-health: a systematic review of systematic reviews (an update). Implement Sci.

[15] Sobieraj S, Krämer NC (2020). Similarities and differences between genders in the usage of computer with different levels of technological complexity. Comput Hum Behav.

